# Application of random forest model to predict the demand of essential medicines for non-communicable diseases management in public health facilities

**DOI:** 10.11604/pamj.2022.42.89.33833

**Published:** 2022-06-02

**Authors:** François Mbonyinshuti, Joseph Nkurunziza, Japhet Niyobuhungiro, Egide Kayitare

**Affiliations:** 1 African Center of Excellence in Data Science (ACE-DS), College of Business and Economics, University of Rwanda, Kigali, Rwanda,; 2 Human Resource for Health Secretariat, Ministry of Health, Kigali, Rwanda,; 3 School of Economics, College of Business and Economics, University of Rwanda, Kigali, Rwanda,; 4 National Council for Science and Technology (NCST), Kigali, Rwanda,; 5 College of Medicine and Health Sciences, University of Rwanda, Kigali, Rwanda

**Keywords:** Non-communicable diseases, essential medicines, random forest, machine learning

## Abstract

**Introduction:**

recent initiatives in healthcare reform have pushed for a better understanding of data complexity and revolution. Given the global prevalence of Non-Communicable Diseases (NCD) and the economic and clinical burden they impose, it is recommended that the management of essential medicines used to treat them be renovated and optimized through the application of predictive modeling such a RF model.

**Methods:**

in this study, a series of data pre-processing activities were used to select the top seventeen (17) NCD essential medicines most commonly used for treating common and frequent NCD. The study focused on machine learning (ML) applications, whereby a random forest (RF) model was applied to predict the demand using essential medicines consumption data from 2015 to 2019 for approximately 500 medical products.

**Results:**

with a seventy-eight (78) percent accuracy rate for the training set and a 71 percent accuracy rate for the testing set, the RF model predicted the trend in demand for 17 NCD essential medicines. This was achieved by entering the month, year, district, and name of the NCD essential medicine. Based on historical consumption data, the RF model can thus be used to predict demand trends. Our findings showed that the RF model is talented to commendably perform as a predicting model.

**Conclusion:**

the study concluded that RF has the ability to optimize health supply chain planning and operational management by boosting the accuracy in predicting the demand trend for NCD essential medicines.

## Introduction

The prediction of essential medicines demand is a critical component and a useful insight for foreseeing future health-care needs, according to today's health supply chain [1]. The World Health Organization´s (WHO) Global Strategy on Digital Health 2020-2025 emphasizes e-Health as a critical component of essential medicine management. Digital health is defined as a multi-functional part of the health system that incorporates digital users and a greater range of smart and linked devices [2]. Because the prediction of future health system demands, such as medicine needs for health service provision and healthcare requirements, is critical for ensuring continuous provision of health services, this area of prediction necessitates special consideration and, in this regard, to construct high-accuracy prediction models, technology-centered applications, such as machine learning, are required [3,4].

### Predictive modeling with machine learning: a random forest model

Health supply chain experts and health-care providers can use predictive modeling and machine learning to optimize their mandate of providing the appropriate services at the right time. It also allows for the analysis of large amounts of data, which can be used to inform interventional procedures and data-driven decisions [5]. The RF predicting model is a behavior analysis and modeling tool based on decision trees. Large data sets generated by today´s supply chain activities, notably in the health industry, can be handled by it. The RF approach examines each case independently and selects the forecast with the most votes as the winner [6]. The RF predicting model is a decision tree-based modeling prediction and behavior analysis tool. It can manage enormous data sets created by supply chain activities, which is very useful in the healthcare industry. The RF model examines each case separately and selects the top-quality forecast based on the predictive model with the most votes [7,8].

The benefits of the RF model stem from the fact that random forests produce the most accurate results of any popular data classification algorithm. The RF technique can also handle large datasets with a set of parameters [9]. The RF is a machine learning model that can handle a wide range of variables quickly, making it ideal for challenging tasks such as health supply chain management. If a class is less common in the data than other classes, data sets can be automatically balanced [10,11]. Using various technology-driven tools and applications to improve supply chain efficiency is a major priority for many businesses. Data analytics and machine learning models can help with supply chain management by anticipating demand and optimizing warehouses for essential medications using the RF model or another analogous machine learning model [12]. Data is a huge plus for those who use it effectively in their line of work, such as those in the health sector. Given the massive amounts of data collected by health supply chain logistics, transportation, and warehousing of essential medicines, the ability to use this data to improve operational performance is crucial [13].

### Brief on non-communicable diseases

Non-communicable diseases (NCDs) kill 41 million people worldwide each year, accounting for 71 percent of all deaths. The most frequent NCDs include cardiovascular illnesses, diabetes, cancer, and chronic respiratory diseases. Low- and middle-income nations account for 85 percent of NCD-related mortality [14]. Moreover, LMICs account for nearly three-quarters of all NCD-related mortality, with 82 percent dying from malnutrition before the age of seventy [15,16]. In recent years, NCDs have lost their status as “rich and noble diseases”, as they can impact any social group on a worldwide scale, with or without boundaries. Because industrialized countries have historically borne the burden of NCDs, they have collected a plethora of disease prevention and control expertise [16]. Noncommunicable diseases (NCDs) look to be the twenty-first century's most serious health and economic burden. They have had a big impact on people's lives and economies, especially in low- and middle-income countries. NCDs are also a barrier to long-term economic gain and advancement [17,18].

Noncommunicable diseases (NCDs) are the leading cause of death and chronic disease in the world, killing more people than all other causes combined. NCDs, which include cardiovascular illnesses, diabetes, cancer, and chronic respiratory diseases, are the leading causes of death [19]. To give such a quick summary of these four types of NCDs, let's start with cardiovascular diseases, which are defined as any disorders that affect the heart and blood vessels, with coronary heart disease, stroke, and peripheral vascular disease being the most frequent [20]. Second, cancers are typically defined as abnormal and uncontrolled cell proliferation (growth) that arises from cells of a specific organ [21]. According to a WHO report from 2018, the most common cancers are lung, colorectal, breast, prostate, stomach, and skin, with lung, stomach, colorectal, liver, and breast cancers accounting for the majority of cancer deaths [22]. Third, chronic respiratory diseases encompass a wide range of illnesses that affect the lungs' airways and other structures. Among them are chronic obstructive pulmonary disease, asthma, and respiratory allergies, as well as pulmonary illnesses [23]. Fourth, type 1 and type 2 diabetes both produce hyperglycemia. Type 1 diabetes develops when pancreatic cells fail to produce enough insulin. Type 2 diabetes causes body cells to be unable to tolerate the amount of insulin produced. It is a long-term condition, but it is also potentially fatal [24].

### The prediction of essential medicine demand for non-communicable diseases

When it comes to health and well-being, the burden of noncommunicable diseases is one of the most pressing challenges, and according to World Health Organization (WHO) data, the availability of corresponding essential medicines is also a major concern for many people in developing countries [25]. Because of the complexities of health supply chain activities, there is no single methodology for forecasting future demand, so a variety of methods need to be applied and evaluated with the intend of assessing their accuracy [26]. There is an urgent need to investigate the practical application of RF models for accurately estimating demand for medical supplies. This study focuses on critical medications used in the management and control of noncommunicable diseases (NCDs). The research presents an overview, analysis, and recommendations for using a random forest model in the health supply chain.

A study conducted in Kirehe District, Rwanda discovered that essential drugs needed to treat hypertension, diabetes, and asthma were frequently overstocked. In addition, the survey found that important drugs were delivered late and insufficiently in relation to the needs of health facilities. It suggested that routine tracking of NCD essential medicines supply levels be improved [27]. As revealed by various studies, the application of machine learning can provide a solution to many challenges identified in health supply chain through a data-driven predictive modeling by improving the accuracy in demand prediction [28,29]. Equally, another study on predicting the demand for essential medicines in Rwanda demonstrated that machine learning models can be used to improve supply chain management in the health sector, where they can serve as the foundation for upgrading the planning process and operational management [30]. Machine learning can assist in estimating demand for medicines used in the treatment and management of noncommunicable diseases (NCDs), hence ensuring the continued supply of essential medicines. However, random forest development and deployment are still required to address the issue of accuracy in predicting NCD essential medicines.

### Study objectives

While the aim of this article is to demonstrate the results of determining future needs for NCD basic drugs based on reported utilization data, specifics objectives for this study were to: describe NCDs essential medicines historical consumption data, to train and test the dataset related to NCDs essential medicines consumption, to develop a RF model for prediction of essential medicines demand. The study is divided into five sections which include the current one as the first section, focusing on the foundational background relating to predictive modeling with machine learning and a brief on no communicable diseases. The section two focuses on the methodological approach, which includes a depiction of settings, sources, types, and exploration of data. The third section discusses data preprocessing and RF predictive modeling techniques, while the fourth section provides a summary of the experimental results and interpretation. Finally, the fifth section discusses the conclusion and recommendations for future research.

## Methods

### Settings

Rwanda, also known as the Land of a Thousand Hills, is a landlocked country that has made universal healthcare access a priority. The country implemented a health development strategy based on decentralized management and district-level coordinated healthcare delivery [31]. As stated in the main country priorities, Rwanda´s health sector is tasked with continuously improving and sustaining population healthcare delivery through the availability of people-centered preventative measures, curative and therapeutic interventions, and rehabilitation programs [32]. Because of the way Rwanda's health supply chain is set up, public health facilities report and request medical supplies through the electronic Logistic Management Information System (eLMIS) tool, which connects the Rwanda Medical Supply (RMS) Ltd central level, RMS branches at the district level, and healthcare delivery points. Essential medicines consumption data, including those used for NCD treatment and management, are included in the reported package. The RMS Ltd is Rwanda's national central medical store, supplying essential medicines as well as NCD-related commodities to district-based RMS branches. To ensure the availability of all necessary health commodities, RMS Ltd collaborates with a faith-based medical store called BUFMAR (*Bureau des Formations Médicales Agréées du Rwanda)* and an approved private medical store called MEDIASOL (Medical & Allied Service Solutions).

### The description and design of the study, sources, types of data

The study is both descriptive and experimental. It used program data generated from after consulting the e-LMIS, an electronic digital tool used in the management of medical products in Rwanda. Data was collected and processed so that it could be used correctly during the predictive modeling process. In this study, the significant task accomplished through time series analysis was the prediction of NCD essential medicine demand [1]. From 2015 to 2019, data generated in the health supply chain, particularly those relating to essential medicines used in the treatment of NCDs, were used in our study. The dataset included a variety of data related to inventory management practices, but our study focused on consumption data that could serve as a basis for demand prediction. NCDs Essential medicine consumption data were extrapolated from Rwanda´s pharmaceutical supply chain using the eLMIS. While the eLMIS tool allows data access at all pharmaceutical supply chain managerial levels, it also allows for the collection over all data at the district level. Our study relied on district-level data that was aggregated at the central level. A dataset examined contained over 500 items used in public health facilities, the majority of which were essential medicines. We only chose seventeen [17] essential medicines that are commonly and mostly used in the management of NCDs, and we concentrated on data related to medicine consumption or distribution. A description of essential medicines considered in our study is presented in Table 1 with reference to the WHO's classification system directed on the anatomical therapeutic chemical (ATC) group of drugs.

**Table 1:** categorization of selected essential medicines

ATC code	NCD´s essential medicines	Pharmacotherapeutic Group
**C09AA01**	Captopril 25 mg tablet	Angiotensin-converting enzyme (ACE) inhibitors
**R03DA05**	Aminophylline 100mg tablet	Bronchodilator consisting of theophylline
**H02AB06**	Prednisolone 5 mg tablet	Corticosteroids
**R03CC02**	Salbutamol 4mg tablet	Selective beta-2-adrenoreceptor agonists
**A10B B01**	Glibenclamide 5 mg tablet	Oral hypoglycemic agents which is a Sulfonyl urea derivative
**C03CA01**	Furosemide 40 mg tablet	High-ceiling diuretic sulfonamides, loop diuretics
**C03AA03**	Hydrochlorothiazide 50mg tablet	Thiazide diuretic
**C08CA05**	Nifedipine 20 mg tablet	Calcium channel blocker with vascular effects (dihydropyridine derivative)
**C03DA01**	Spironolactone 25 mg tablet	Potassium-sparing Agents
**A10BA02**	Metformin 500 mg tablet	Blood glucose lowering drugs. Biguanide oral hypoglycemic agents
**C01CA26**	Ephedrine 50mg/ml injection	Adrenergic and dopaminergic agents
**A10AC01**	Insulin human 100ui/ml injection (Humulin® 100 IU/ml)	Insulins and analogues for injection, intermediate-acting
**L02BA01**	Tamoxifen citrate 20mg tablet	Anti-estrogens
**N02AA01**	Morphine 10 mg/ml injection	Opioids, natural opium alkaloids
**M04AA01**	Allopurinol 100mg tablet	Antigout preparations inhibiting uric acid production
**R03BA01**	Beclomethasone 250mcg cfc	Glucocorticoid
**C09AA02**	Enalapril 5 mg tablet	Angiotensin converting enzyme (ACE) inhibitor

**Figure 1:**
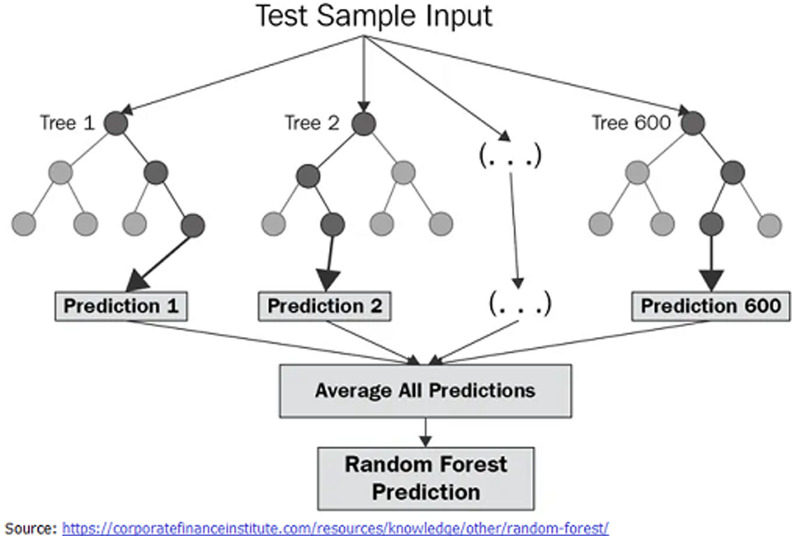
description of RF model

As shown in Table 1, data from 2015 to 2019 for each quarter were combined and used in this study. Meant for preprocessing, data related to seventeen NCD essential medicines were inputted into “DataFrame” by using the function read excel from pandas´ library. To reduce the number of variables that are unsuitable for our tasks, only five stakes (variables) were retained. The variables retained are the quantity consumed, the name of the essential medicines consumed, the consumer districts (which includes all consumption from district-based health facilities), the year and month of consumption. Each character hindering data processing to the “DateTime” type, and data entry errors such as the indication “none” in a year column, were cleaned up. However, none of the lines, nor those of the characters were altered. We had a large number of observations without districts, so we inferred district data within the same district using data from health facilities. Because there were no decimals, the quantity of essential medicines purchased was converted to an integer, and absolute functions were used to minimize unwanted values. Despite the fact that the dataset contained nearly 500 medical items, our study focused only on seventeen of them, which are the most commonly used NCD essential medicines. Figure 1 depicts the quantity and frequency of consumption for these 17 items, which are used to build a model for predicting the future demand of NCD essential medicines.

### Data description and exploration

According to Table 2, which describe the consumption of essential medicines for non-communicable disease by quantity and frequency (2015-2019), the first 3 most consumed NCD essential medicines by count are aminophylline 100mg tablet b/1000 with a frequency of 19,475, the second consumed medicine is salbutamol 4mg tablet b/1000 with a frequency of 17,178, the third consumed medicine is prednisolone 5 mg tablet b/1000 item at frequency of 17,091. Similarly, considering the quantity amount of consumption, the top three first NCDs essential medicines most consumed, are aminophylline 100mg tablet b/100 in an amount equal to 26,839,457 tablets, followed by salbutamol 4mg tablet in an amount equal to 25,753,669 tablets, and prednisolone 5 mg tablet b/1000 with an amount equivalent to 23,598,175. The information captured in Table 2 illustrates how essential medicines for NCDs were consumed by quantity and frequency (2015-2019).

**Table 2:** consumption of essential medicines for NCDs by quantity and frequency (2015-2019)

#	Name of Essential Medicines	Quantity	Frequency
**1**	Aminophylline 100mg tablet b/1000	26,839,457	19,475
**2**	Salbutamol 4mg tablet b/1000	25,753,669	17,178
**3**	Prednisolone 5 mg tablet b/1000	23,598,175	17,091
**4**	Captopril 25 mg tablet b/100	15,140,860	9,446
**5**	Furosemide 40 mg tablet b/1000	13,464,774	7,873
**6**	Glibenclamide 5 mg tablet b/1000	11,641,185	6,786
**7**	Insulin hum 100ui/ml Lente inj b/1	5,756,901	3,705
**8**	Hydrochlorothiazide 50mg tablet b/1000	3,435,412	2,910
**9**	Spironolactone 25 mg tablet b/100	2,348,623	2,626
**10**	Ephedrine 50mg/ml injection b/100	553,424	2,439
**11**	Beclomethasone 250mcg cfc/vial	107,040	2,366
**12**	Morphine 10 mg/ml injection b/100	95,178	1,086
**13**	Nifedipine 20 mg tablet b/1	81,422	786
**14**	Metformin 500 mg tablet b/28	53,250	374
**15**	Allopurinol 100mg tablet b/1	33,780	94
**16**	Tamoxifen citrate 20mg tablet b/1	32,381	84
**17**	Enalapril 5 mg tablet b/28	21,824	40

### Description of the random forest model

**Figure 2:**
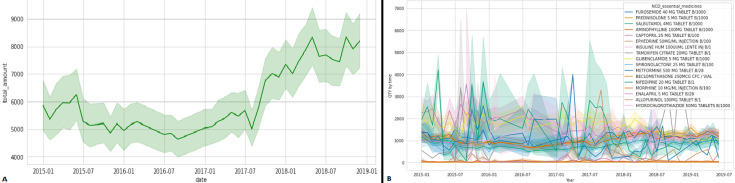
A) the trend of quantity consumed for all combined NCD essential medicines; B) the trend of quantity consumed for each individual NCD essential medicines

As described in Figure 2 relating to RF techniques, during training, the RF model is composed of an ensemble learning method regression and other tasks that operate by customizing and making a bigger number of decision trees. The mean or average prediction of the individual trees is handed back for regression tasks. RF algorithm with 25000 estimators, 15 maximum depth, 12 maximum features, 8 minimum critical sample, and RF state set to zero was used in the context of our study.

### Experimental exploration of RF model and evaluation

Machine learning is a game-changing topic in the field of business information technology. With advances in technology and a preference for computer-based business management, machine learning models can extract actionable insights from historical data and mimic the practical way of a human being to forecast various aspect and future business trend [33]. This study used RF as a ML model, which focuses on forecasting demand in the health supply chain. After training the model, its predictions were compared to the true target on the training data. The model was also tested on new data that had not been used in training, and its accuracy was determined using root mean square error (RMSE) and R-square (R2). RMSE: The Root Mean Square Error (RMSE) is a standard tool for determining a model´s error in predicting quantitative data. In the RMSE score, errors are squared before being averaged. As a result, larger errors are given more weight. Logically, RMSE measurement considers that large errors can have a significant impact on how the model predicts. Such a feature is useful in many mathematical computations because it avoids calculating the absolute value of the error. The lower the value of this metric, the better the model´s performance. R-square (R2): the coefficient of determination, also known as R-squared or R2 in the scientific literature is a metric that illustrates how well a model fits a given dataset. It exemplifies how closely the regression line (the plotted predicted values) corresponds to the real data. Its values range from 0 to 1, with 0 asserting that the model does not fit the data and 1 designating that the model predicted values fully fits the real data.

### Ethical considerations

Research used historical program data from supply chain management and related datasets in Rwanda Health system such existing electronic data management tools such as e-LMIS (electronic Logistic Management Information System). The National Health Research Committee authorized the research (Ref No: NHRC/2020/PROT/015), and the Ministry of Health (Rwanda) provided a research collaborative notice.

## Results

### The trend of essential medicine consumption by time

As indicated in Figure 2A, the quantity consumed of NCD essential medicines increased from July 2017 and maintain the pic up to June 2019. This may be associated to the fact that in that period Rwanda has prioritized three intervention options to manage NCDs, including community action and engagement as an important component of changing behaviors and increasing early detection. Another intervention focuses on prevention and management of NCD risk factors such as poor diet, excessive alcohol consumption, and smoking. Figure 2B illustrates the individual trend in consumption for each of the seventeen NCD essential medicines considered for the study and shows that the top five most commonly used essential medicines are furosemide 40 mg tablet, salbutamol 4 mg tablet, prednisolone 5 mg tablet, aminophylline 100 mg tablet, and captopril 25 mg tablet. Figure 1 and Figure 2 show a shift in trends that may be related to countries' efforts to combat non-communicable disease by expanding access to care at all levels, including primary care. As a result, high-quality NCD care and management have been prioritized at all levels of care delivery [34].

### Geographical distribution of NCD essential medicines consumption in Rwanda

**Figure 3:**
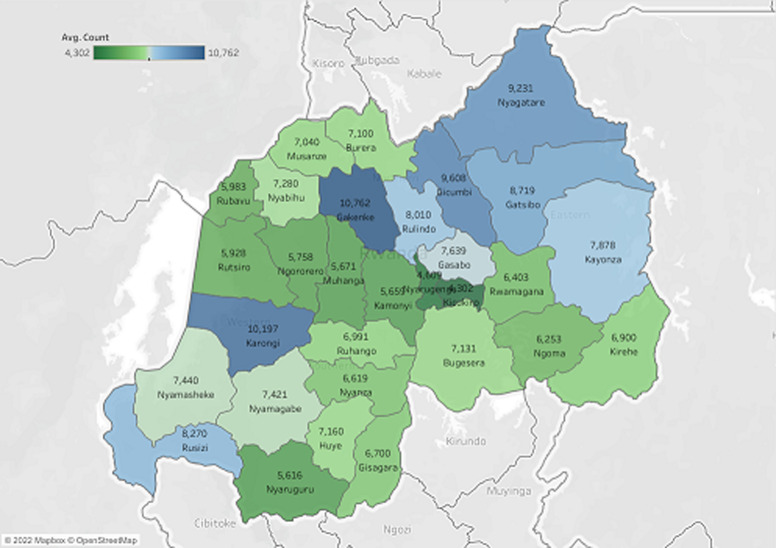
mapping the consumption of NCD essential medicines stratified by district

In accordance to Figure 3, ten districts in Rwanda, including Kigali City, out of 30 were ranked as having the highest consumption of NCD essential medicines. Nyagatare, Gatsibo, Kayonza, Gicumbi, Rulindo, Gakenke, Burera, Karongi, and Rusizi are the concerned districts. Also, according to this map, Kigali City which comprise Gasabo, Nyarugenge, and Kicukiro districts, is among the lowest consumers of NCD essential medicines. From Figure 3 observations, Low consumption of NCD essential medicine in Kigali City may be explained by a large number of cases managed in private clinics and thus receiving medicines in private pharmacies. Most common non-communicable diseases requiring the prescription of essential medicines are managed at primary healthcare level, and frequently in rural health facilities.

### Application of predictive modeling using RF model: test and train

To learn its generalizability to new data, the model was trained on one set of observations and tested on a different set of observations using machine learning. The data was divided into two categories. The train was operational from January 2015 to June 2018, with test has taken the period from July 2018 to June 2019. We divided the data by year and month because we want to predict the amount of a particular NCD essential medicine on a monthly basis. Usually, the greater the degree of ambiguity in outlooks, the greater the level of discrepancy in time series prediction [35,36]. Following that, considering the type and volume of available past consumption data, the RF model should be designated as a suitable technique from among the various viewing platforms that can be used in the predictive model. Relating to Table 3, which presents summary statistics on the amount of NCD essential medicines consumed by the training and testing groups, provides a summary of the amount of NCD essential medicines consumed in both training and testing sets before categorizing the data by year, month, type of NCD essential medicine, and district. In fact, for any type of NCD essential medicine, we have monthly district-level consumption data. There were 73,066 observations on the train set, with an average of 992.76 essential medicines consumed, a standard deviation of 1370.16, a median of 495, a minimum value of 4, and a maximum value of 8200, and a standard deviation of 1370.16, a median of 495, a smaller value of 4, and highest amount of 8200. There were 11659 observations in total, with a mean of 6208.58, a standard deviation of 7241.64, a median of 3765, a threshold of 4 and a peak of 8186. The test set included 28,680 observations, with a mean of 1069.83 for the quantity of essential medicines used for NCDs, a standard deviation of 1387.78, a median of 570, a threshold of 4, and a peak of 5602. Data has been grouped and the total number of observations was 3733, with a mean of 8187.30, a standard deviation of 8931.23, a median of 5602, the lowest value of 4 and an upper limit of 57568.00.

**Table 3:** summary statistics on the amount of NCD essential medicines consumed by the training and testing groups

Set	Variable	Count	Mean	SD*	Median	Min	Max
**Training**	Amount	73,066.00	992.76	1,370.16	495.00	4.00	8,200.00
	Grouped/Total Amount	11,659	6,208.58	7,241.64	3,765.00	4.00	8,186.00
**Testing**	Amount	28,680.00	1,069.83	1,387.78	570.00	4.00	5,602.00
	Grouped/Total Amount	3,733.00	8,187.30	8,931.23	5,602.00	4.00	57,568.00

*SD= Standard deviation

## Discussion

**Figure 4:**
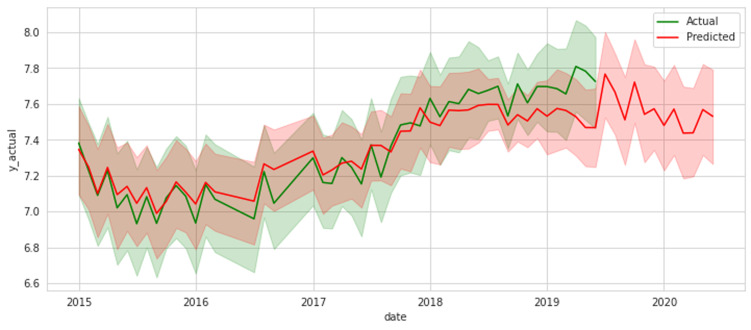
the use RF model for prediction of NCD essential medicines demand (projected needs) versus actual consumed quantity on monthly basis

According to Table 4 presenting the RF Model results, the root mean squared error of RF is 1.137 on a training set, 1.23 on a testing set, and the R-square of RF is 0.78 on a training set, 0.71 on a testing set. The RF model can accurately predict consumed item for a given month, type of essential medicines, and district from these values at a level of 78% on a training set and 71% on a testing set based on these values. As a result, the RF model has a satisfactory fitness, with only a 5% difference between train and test prediction. We could confidently state that it would generalize to a new dataset. As shown in Figure 4, the RF model was applied for the prediction of NCD essential medicines quantity versus actual consumed quantity. In this viewpoint, one of our study´s key experimental results is evidence that the predicted values released by the RF model were approximately similar to the real data (only minor variation were observed). Based on this, RF can be reported as having the capability to predict the trend of demand for essential NCD medicines.

**Table 4:** presentation of random forest model experimental results

Model	Set	RMSE	R-square
**Random Forest**	Train	1.137	0.78
	Test	1.23	0.71

Our research article described a predictive modeling using machine learning in which the RF model was used to forecast the demand for essential medicines used in the management and treatment of essential medicines. The model construction process is comprised of the following steps, each of which clearly achieves the desired goal: First, the data was extracted from real-world data, specifically data related to the management of Rwanda´s health supply chain. The research used data covering a five-year period, and the data was clearly explained through visualization. The second task was to fix a training dataset containing data from January 2015 to June 2018, as well as a testing dataset containing data from July 2018 to June 2019. At this point, the distribution of data was recognized, as was a clear thoughtful of their description. Third, following the design and development of RF model, good results were observed, indicating an acceptable range of prediction accuracy. Our findings are consistent with and supported by evidence from other studies, such as one conducted by Dash *et al*. on the integration of analytics and machine learning approaches for biomedical and healthcare data [11], and another by Ramos *et al*. on the use of data mining approaches with spatial characteristics to improve medication demand forecasts [25]. It has been revealed in numerous scenarios that forecasting the needs for healthcare supplies and inventory management are still significant issues in health system management today. As a result, our research confirmed the use of an RF model to forecast the demand for NCD-related essential drugs and therefore it should remain a top focus in order to keep the health-care system running smoothly.

## Conclusion

The RF model has been developed and tested with an R-square accuracy of 0.78 on a training set and 0.71 on a testing set and the experimental results pointed out the lowest error value upon developing the RF model. The research focused on flagging the evidence resulting from forecasting future needs for NCD essential medicines based on historical consumption data. Subsequently, the model fitness has been shown to optimize prediction because the difference between train and test prediction is only 5%, and we can conclude that it is generalizable to new datasets. Based on the observations from this study, it is undoubtedly recommended to extend future research to sustainably rationalize the use of machine learning applications in other supply chain activities such as manufacturing, packaging, distribution or transportation, inventory management, demand planning, warehousing, and customer service to name few. Furthermore, future study may look into the possibilities of using the RF model to promote inexpensive access to vital medicines in other contexts, such as infectious disease control and management.

### What is known about this topic

- Machine learning has emerged as one of the most applied technologies in various fields due to its multiple capabilities that are absolutely essential to business and institutional success, such as making accurate forecasts and recognizing patterns;
- Random Forest is one of the Machine Learning models that has demonstrated potential use in predicting supply chain demand;
- Decision-makers can efficiently monitor logistics while avoiding supply disruptions such as shortages, out-of-stock, overstock, and expiries, to name few by adopting the application of machine learning techniques.

### What this study adds

- As the supply of essential medicines is constantly evolving and shifting as a result of the technological revolution, the Random Forest model is regarded as useful and effective model for managing the digital health supply chain, notably for predicting essential medicines demand;
- There is thus a need to explore Random Forest, which is on of Machine Learning models for particularly predicting the demand of essential medicines, required for public health facilities to function properly and deal with the evolving challenge associated with non-communicable diseases;
- The Random Forest model is enabled to contribute for sightseeing more accuracy in demand prediction of non-communicable diseases related essential medicines.
